# Insulin Treatment Reduces Susceptibility to Atrial Fibrillation in Type 1 Diabetic Mice

**DOI:** 10.3389/fcvm.2020.00134

**Published:** 2020-08-12

**Authors:** Zahra Maria, Allison R. Campolo, Benjamin J. Scherlag, Jerry W. Ritchey, Véronique A. Lacombe

**Affiliations:** ^1^Department of Physiological Sciences, Oklahoma State University, Stillwater, OK, United States; ^2^Harold Hamm Diabetes Center, University of Oklahoma Health Sciences Center, Oklahoma City, OK, United States; ^3^Department of Internal Medicine, University of Oklahoma College of Medicine, Oklahoma City, OK, United States; ^4^Department of Veterinary Pathobiology, Oklahoma State University, Stillwater, OK, United States

**Keywords:** glucose transporters, arrhythmias, glycogen, metabolism, diabetes, atria

## Abstract

Diabetes has been identified as an independent risk factor for atrial fibrillation (AF), the most common chronic cardiac arrhythmia. Whether or not glucose and insulin disturbances observed during diabetes enhance arrhythmogenicity of the atria, potentially leading to AF, is not well-known. We hypothesized that insulin deficiency and impaired glucose transport provide a metabolic substrate for the development and maintenance of AF during diabetes. Transesophageal atrial pacing was used to induce AF in healthy, streptozotocin-induced insulin-deficient type 1 diabetic, and insulin-treated diabetic mice. Translocation of insulin-sensitive glucose transporters (GLUTs) to the atrial cell surface was measured using a biotinylated photolabeling assay in the perfused heart. Fibrosis and glycogen accumulation in the atrium were measured using histological analysis. Diabetic mice displayed mild hyperglycemia, increased duration and frequency of AF episodes vs. age-matched controls (e.g., AF duration: 19.7 ± 6.8 s vs. 1.8 ± 1.1 s, respectively, *p* = 0.032), whereas insulin-treated diabetic animals did not. The translocation of insulin-sensitive GLUT-4 and -8 to the atrial cell surface was significantly downregulated in the diabetic mice (by 67 and 79%, respectively; *p* ≤ 0.001), and rescued by insulin treatment. We did not observe fibrosis or glycogen accumulation in the atria of diabetic mice. Therefore, these data suggest that insulin and glucose disturbances were sufficient to induce AF susceptibility during mild diabetes.

## Introduction

Atrial fibrillation (AF) has been identified as the most common chronic abnormal heart rhythm, although AF can also be paroxysmal. Approximately 33 million people (2.2 million in the United States alone) suffer from this condition and it is predicted to double by 2050 (1). AF has been associated with 4–5-fold increase in the risk of ischemic stroke, a 3-fold increase in heart failure, and a 40–90% increase in mortality (2–4). Identification of the underlying pathophysiological mechanisms is required for the development of novel therapeutic strategies for AF. While diabetes is an epidemic disease currently affecting 9% of the US population, and has been identified as one of the most important risk factors for AF (5, 6), the mechanisms underlying increased AF propensity during metabolic diseases are not well-known. For instance, it has been reported that diabetes increases the odds of developing AF by 1.4 and 1.6-fold in male and female patients, respectively (4). In population-based cohort studies, it was reported that diabetes was responsible for a ~30–40% increased risk of AF and that the risk of AF is enhanced with increased duration of diabetes (6–8). However, a causative link between diabetes and AF is not well-established and whether a metabolic substrate underlies AF remains elusive.

In addition to structural-electrical remodeling, altered glucose metabolism may directly play important and interrelating roles in the pathogenesis of AF in diabetic patients (8). This is germane to the fact that some risk factors of AF included the duration of diabetes and level of glycemic control (9). For instance, in a population-based study (out of 11,140 patients), 7.6% of diabetic patients were diagnosed with AF at baseline (10). Furthermore, it has been reported that the risk of AF in individuals with insulin-deficient (type 1 and advanced type 2) diabetes increased with worsening glycemic control and periods of hypoglycemia (11–13). Similarly, severe hypoglycemia-induced fatal cardiac arrhythmias were enhanced in diabetic rats with insulin deficiency (14). Recently, we demonstrated that long term high-fat diet-induced insulin resistance enhanced the vulnerability of AF induction in a rodent model (15). Taken together, the results of these studies suggest that disturbances in glucose and insulin could enhance the arrhythmogenicity of the atrium, which contains the pacemaker of the heart, potentially leading to AF (6, 9, 16). However, it remains to be determined whether alterations in myocardial glucose metabolism plays a direct role in the pathogenesis of AF.

Since cardiac myocytes contract for each heartbeat, the metabolic demands in the heart are high. In order to sustain this high energy demand, cardiac myocytes have a high rate of glucose utilization, despite the ability of the myocardium to use other substrates (17). Glucose uptake from the blood into the cells is tightly regulated by a specialized family of glucose transporters (GLUTs) (18). So far a total of 14 GLUT isoforms have been identified and distributed into 3 classes (15, 19, 20). The translocation of GLUT4 to cell surface, the major insulin-sensitive isoform, which precedes glucose uptake and oxidation, is mediated by AS160 phosphorylation and rabGTP activation downstream of the insulin-signaling pathway (21), Importantly, we recently reported that translocation of insulin-sensitive GLUT-4 and -8 to the cell surface was significantly impaired in the atria of insulin deficient type 1 diabetic mice, which was restored following *in vitro* insulin stimulation (19). These findings suggested that *in vivo* insulin treatment could possibly restore alterations in atrial glucose transport during diabetes.

Insulin stimulates both glucose transport and glycogen synthase activity in striated muscle. Once glucose is inside the muscle cell, it is transformed to G-6-P through the activation of hexokinase and becomes trapped in the muscle cell. Under the action of glycogen synthase, G-1-P is converted to glycogen (22). Although the glycogen pool in the heart is relatively small and has a relatively rapid turnover (17), increased accumulation of glycogen has been reported in the myocardium of diabetic subjects and in a goat model, which may induce electrophysiological conduction blockade (23–27). However, whether glycogen accumulation leads to electrophysiological disturbances and AF remains to be elucidated.

Although 10–25% of AF patients are diabetic (16, 28), there are almost no studies that have investigated whether interventions to reduce the hyperglycemic burden or dysglycemia during diabetes could reduce the AF burden. As many of these diabetic patients suffer from type 2 diabetes in particular, we have recently identified that insulin dysregulation complicated by obesity significantly increased the propensity toward developing AF (15). However, it is unknown if chronic hyperglycemia alone (without the confounding variable of obesity) also leads to increased AF susceptibility. Given the association between hyperglycemia and AF, we investigated the role of insulin deficiency toward susceptibility and propensity of AF in type 1 diabetic animals. In addition, we evaluated the role of insulin treatment in reducing AF induction. We hypothesized that (1) insulin-deficient type 1 diabetic mice will have greater propensity for AF and (2) insulin treatment will rescue alterations in glucose transport in the atria of insulin-deficient type 1 diabetic mice, and thereby reduce AF vulnerability.

## Methods

### Induction of Insulin-Deficient Diabetes

**Figure 1:**
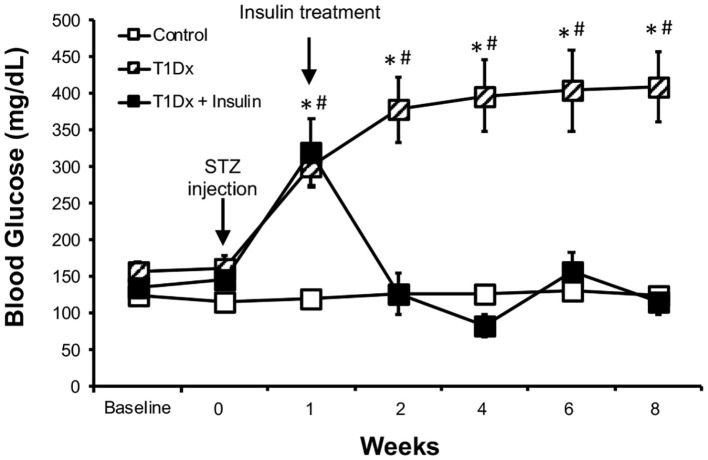
Streptozotocin-induced hyperglycemia was rescued by *in vivo* insulin treatment in the treated diabetic group. Mean ± SE of fasted serum blood glucose levels of untreated type 1 diabetic (T1Dx, *n* = 12), insulin-treated type 1 diabetic (T1Dx+Ins, *n* = 9), and control animals (*n* = 12/group). #*P* < 0.05 vs. Baseline, *P < 0.05 vs. insulin-treated T1Dx. Statistical test: 2-way repeat measure ANOVA.

All procedures were done according to the NIH guidelines and approved by the Oklahoma State University Institutional Animal Care and Use Committee (animal protocol # VM-12-3). Male FVBN/J mice were obtained from Jackson Laboratory (Bar Harbor, ME) at the age of 8–10 weeks. The animals were randomly allocated to one of the following 3 groups (*n* = 9–12/group): control, insulin-deficient (type 1) diabetic (T1Dx), and insulin-treated diabetic (T1Dx+Insulin). Insulin deficiency was induced for 8 weeks by 3 consecutive low-to-mild doses (29) of intraperitoneal injection of streptozotocin (STZ, 65–95 mg/kg, at 48 h intervals), while the control group (control) only received injections of the vehicle (citrate buffer) (19, 30, 31). Venous blood glucose concentration (from facial vein) was measured at baseline and every week on mice fasted for 6 h using a glucometer (Bayer Contour, Tarrytown, NY). Diabetes was confirmed when fasted venous blood glucose concentration was ≥200 mg/dl (29, 32). Once hyperglycemia was confirmed, a subgroup of the diabetic mice was treated with exogenous insulin (T1Dx+Insulin) for 7 weeks by the insertion of a subcutaneous insulin pump (ALZET mini osmotic pump, HumulinR, 0.5 U of insulin/per mouse/per day, Model 1004; weight of filled pump 0.41 g, dispensed volume 0.5 ml). The osmotic insulin pumps were inserted the day after diabetes was confirmed (on week 1). At this dose of insulin, we did not observe hypoglycemia in treated diabetic animals. While all animals used in this study were included in the *in vivo* data (Figure 1), not all animals were included in the *in vitro* experiments due to the small size of the mouse atria or due to potential confounding variables secondary to the AF induction experiments.

### Induction of Atrial Fibrillation

Atrial fibrillation was induced in control, untreated and treated diabetic animals via closed chest trans-esophageal atrial pacing under anesthesia (ketamine/xylazine intraperitoneal injection; Dose: 87.5 mg/kg ketamine; 12.5 mg/kg xylazine). Atrial pacing was achieved by inserting a 2.2 Fr 6-polar catheter through the esophagus and by positioning it near the left atrium, as previously described (15, 33). Body surface ECG was recorded via dual subcutaneous ECG leads with a telemetric data communication to AliveCor iphone application (AliveCor, Inc., San Francisco, CA). In order to induce AF, burst pacing, using an AC-powered stimulator (Grass Instruments Company, Quincy, MA), was applied for 10 s at 2 different frequencies: 20 Hz (1,200/min) and 40 Hz (2,400/min) twice, for a total of four bursts per animal.

AF was defined as no discernible P-waves and irregular R-R intervals. Duration (in seconds), frequency (number of incidences per animal) of AF, atrial tachycardia (i.e., increased heart rate without defined P waves), sick sinus syndrome (i.e., long sinus pauses and slow recovery, without associated tachycardias), and tachy-brady syndrome (i.e., intermittent sinus pauses and intermittent tachycardia) were recorded from the surface ECG traces, as previously described (15). Atrial tachy arrhythmia included atrial fibrillation, atrial tachycardia, sick sinus syndrome, and tachy-brady syndrome. ECG traces were analyzed by an operator who was blinded to the treatment groups.

### Protein Extraction

Total and membrane-enriched protein extracts were obtained from fresh or frozen pooled left and right atrium, as previously described (15, 19, 30, 31). Briefly, total protein extracts were obtained by incubating atrial tissue in RIPA lysis buffer (Thermo Fisher Scientific) containing 0.2% protease inhibitor for 1 h at 4°C. Following centrifugation at 3,000 g for 30 min at 4°C, the supernatant was collected and stored at −80°C for analysis. In order to obtain membrane enriched extracts, atrial tissue was first homogenized in a buffer containing 210 mM sucrose, 40 mM NaCl, 2 mM EDTA, 30 mM HEPES, and 2% protease inhibitor cocktail (Sigma, St. Louis, MO). The homogenate was subsequently incubated in a buffer containing 58 mM sodium pyrophosphate and 1.17 mM KCl. The membrane fraction was recovered by centrifugation at 40,700 g for 90 min at 4°C. The collected pellet was re-suspended in a buffer containing RIPA and 0.2% protease inhibitor and incubated for 1 h, following which the samples were centrifuged at 3,000 g for 30 min at 4°C. The supernatant was stored at −80°C for further analysis.

### Quantification of Active Cell Surface GLUT Expression

Immediately following excision, the heart underwent Langendorff perfusion as previously described (15, 19, 30, 31). The impermeant biotinylated photolabeling reagent (bio-LC-ATB-BGPA, Toronto Research Chemicals, ON, Canada) was perfused through the aorta of the intact heart for 1 min. The intact heart was then incubated in the photolabeling reagent for 15 min in the dark at 4°C. The atria were exposed to UV using a Rayonet photochemical reactor (340 nm, Southern New England UV) which ensured the cross-linkage between the photolabeling compound and the extracellular binding site of GLUT protein. Recovery of the photolabeled cell-surface fraction of GLUTs from total membrane extraction was achieved using streptavidin bound 6% agarose beads, to allow separation of non-cell-surface GLUTs (“unlabeled” or intracellular fraction that remains in the supernatant) from cell-surface GLUTs (“labeled” or membrane bound fraction). Proteins from the labeled fraction were quantified by densitometry relative to the positive control, as described below (15, 19, 30, 31).

### Protein Extraction and Western Immunoblotting

Briefly, equal amounts of protein (5–20 μg) were resolved in an 8–12% SDS-polyacrylamide gel and electrophoretically transferred to a polyvinyl-idine fluoride membrane (BioRad), as previously described (15, 19, 30, 31). After incubating in blocking buffer (5% non-fat dry milk or 2% goat serum albumin in phosphate buffered saline with 0.1% Tween-20) for 1 h, membranes were incubated with primary antibodies overnight (anti-human GLUT4, 1:750, AbD Serotec; anti-human GLUT8, 1:500; 1:1000, Cell Signaling; rabbit anti-MMP9, 1:1000, EMD Millipore and rabbit anti-TGFβ-1, 1:300, Abcam Antibodies) followed by a 1 h incubation of appropriate secondary antibodies conjugated to horseradish peroxidase (polyclonal goat anti-rabbit; 1:2500, GE Healthcare). Primary antibodies were chosen based on their 100% sequence homology with the protein of interest in rodents, and validated against a positive control (i.e., tissue, peptide). Antibody-bound transporter proteins were quantified by enhanced chemiluminescence reaction (KPL). Band density was quantified using GelPro Analyzer (Media Cybernetics). The data was expressed relative to appropriate controls (i.e., atrial tissue from healthy mice). Equal protein loading was confirmed by reprobing each membrane with calsequestrin monoclonal IgG (Thermo-Scientific PA1-903, 1:2500, polyclonal rabbit anti-dog).

### Histochemical Procedures

Glycogen accumulation was assessed by Periodic acid-Schiff (PAS) staining, with and without diastase digestion, from paraffin-embedded sections. Atrial fibrosis was assessed from Masson's trichrome stained tissue sections as previously described (23, 24).

### Statistical Analysis

Statistical power was calculated for all quantifications, and Shapiro-wilk and Levene's test were used to assess the normality and homogeneity of data, respectively (SigmaStat 4.0, Jandel Scientific). For blood glucose concentrations, differences between means were assessed using repeated measured 2-way ANOVA (duration and treatment). For all *in vitro* experiments (control, untreated and treated diabetic groups), differences between means were assessed using 1-way ANOVA with Student Newman Keuls *post-hoc* test as appropriate. Correlations were performed using Spearman rank correlation analyses. Statistical significance was defined as *P* < 0.05. Data was reported as mean ± standard error (SE).

## Results

### Validation of the Animal Diabetic Model

To investigate the role of diabetes in the development of atrial fibrillation, we used an insulin-deficient diabetic animal model (type 1 diabetes, T1Dx). Diabetic mice displayed mild hyperglycemia for the 8-week period following the streptozotocin injection, while the control group remained euglycemic (diabetic: 386 ± 180 mg/dL vs. control: 125 ± 15 mg/dL at 8 weeks after STZ or placebo injection, respectively, Figure 1). *In vivo* insulin treatment rescued hyperglycemia and insulin-treated diabetic mice maintained normo-glycemia (116 ± 61 mg/dL), following the insertion of the continuous release subcutaneous osmotic insulin pumps.

### *In vivo* Insulin Treatment Reduced the Frequency and Duration of AF Episodes in Diabetic Mice

**Figure 2:**
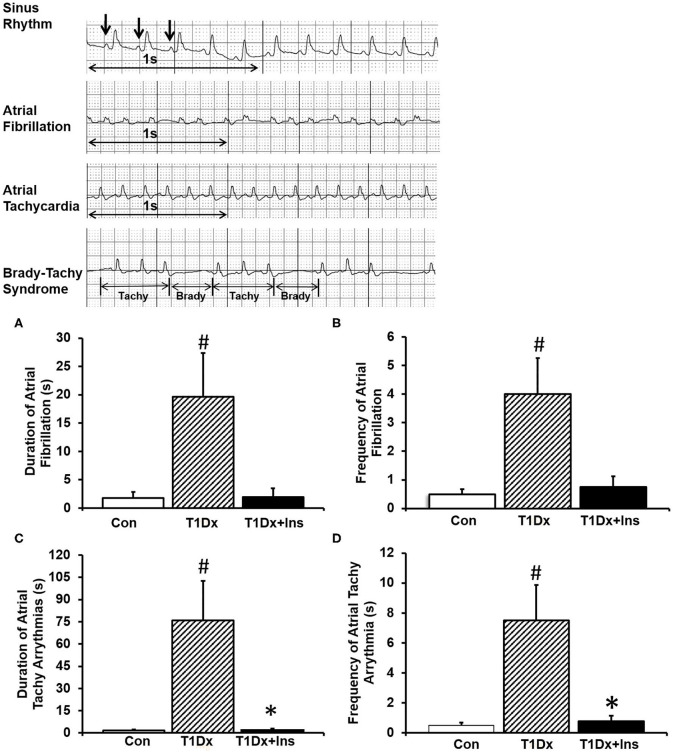
Insulin-deficient type 1 diabetic (T1Dx) animals were more vulnerable to atrial fibrillation, which is rescued by *in vivo* insulin treatment (T1Dx+Ins). **(A)** Longer duration and **(B)** greater frequency of induced atrial fibrillation in T1Dx animals. Top panels: Representative ECG Traces; bottom panels: Mean ± SE of duration (seconds) and frequency (incidence/animal) of AF episodes. **(C)** Longer duration and **(D)** greater frequency of induced atrial tachy-arrhythmias in T1Dx animals, which were rescued in T1Dx+Ins group. Methods: transesophageal atrial pacing; Control: *n* = 8, T1Dx: *n* = 8, and T1Dx+Ins: *n* = 4; #*P* < 0.05 vs. Control, *P < 0.05 vs. T1Dx. Statistical test: 1-way ANOVA.

Atrial tachy-arrhythmias were recorded and analyzed from surface ECG traces following each burst of atrial pacing. T1Dx animals exhibited longer duration and greater frequency of AF episodes (by 1,115 and 800%, respectively, *P* = 0.050 and *P* = 0.028, respectively, Figures 2A,B), which was rescued by *in vivo* insulin treatment. Importantly, AF was observed in 70% of T1Dx animals (7 out of 10) compared to 37% in the control (3 out of 8) and 50% in T1Dx+Ins (2 out of 4) groups. The total duration of AF episodes was 19.6 ± 7.7 s in diabetic vs. 1.8 ± 1.1 s in age-matched control group (*p* = 0.032) and 1.9 ± 1.5 s in age-matched insulin-treated group (*p* = 0.137), with duration ranging from 2.8 to 62.1 s (Figures 2A,B). In addition, longer duration and greater frequency of atrial tachy-arrhythmias (including atrial fibrillation, atrial tachycardia, and brady-tachy syndrome) were also recorded in T1Dx animals (greater by 4,461 and 1,500%, respectively, *P* = 0.002 and *P* = 0.002, respectively, Figures 2C,D). The vulnerability to atrial tachy-arrhythmias was also significantly reduced in insulin-treated animals (*P* = 0.016 and *P* = 0.016 vs. T1Dx for duration and frequency, respectively). The heart rate of diabetic mice during AF episodes (bpm) was significantly higher compared to heart rate prior to AF induction (240 ± 20 and 202.5 ± 6.2 bpm, respectively, *P* = 0.03). We further reported a significant linear correlation between the duration and frequency of AF (y = 0.050x – 1.2721, *r*_s_ = 0.547, *P* = 0.013; y = 0.0126x – 0.6538, *r*_s_ = 0.562, *P* = 0.009), and the duration and frequency of atrial tachy-arrhythmias (y = 0.4313x - 57.443, *r*_s_ = 0.706, *P* < 0.001; y = 0.0399x – 4.5158, *r*_s_ = 0.702, *P* < 0.001, respectively) with blood glucose levels of healthy and diabetic (untreated and treated diabetic) mice. Together, these findings indicate higher inducibility of atrial tachy-arrhythmias (including AF) in the untreated T1Dx animals which was rescued in the insulin-treated diabetic group.

### *In vivo* Insulin Treatment Rescued the Alterations in GLUT Protein Expression and Trafficking in the Atria of Diabetic Animals

**Figure 3:**
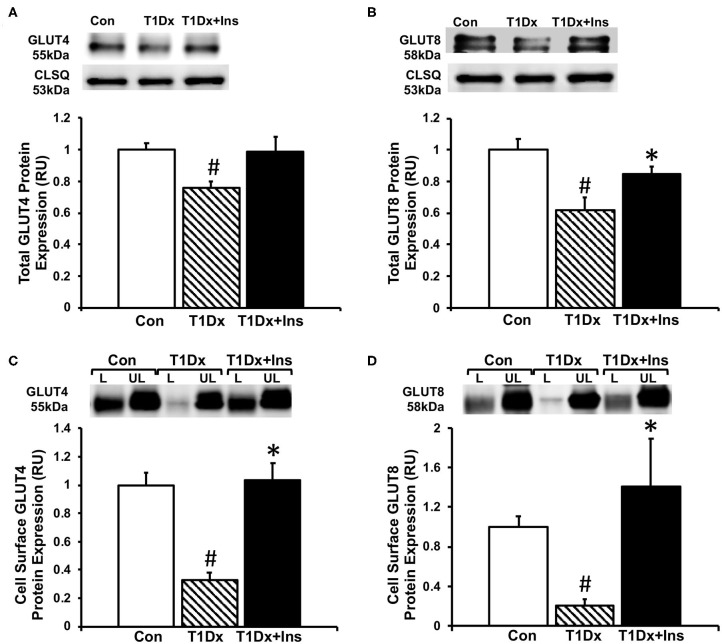
Alterations in glucose transporter (GLUT) trafficking in the atria of insulin-deficient type 1 diabetic (T1Dx) animals were rescued by *in vivo* insulin treatment. Total expression of **(A)** GLUT4 and **(B)** GLUT8 in the atria of the T1Dx, insulin-treated type 1 diabetic (T1Dx+Insulin), and control (Con) animals. Top panels: representative Western blot from total lysate; loading control: calsequestrin (CLSQ). Bottom Panels: Mean ± SE of total GLUT protein content, normalized to calsequestrin (values normalized to respective controls). Methods: Western blotting. Control: *n* = 4, T1Dx: *n* = 3, and T1Dx+Ins: *n* = 4. Atrial cell surface **(C)** GLUT4 and **(D)** GLUT8 protein expression in T1Dx, T1Dx+Insulin, and control animals. Top panels: representative Western blot. Bottom Panels: Mean ± SE of cell surface GLUT protein content (values normalized to controls); Control: *n* = 4, T1Dx: *n* = 4, and T1Dx+Ins: *n* = 5. Methods: biotinylated photolabeling technique in the intact perfused mouse heart. L, Labeled (cell surface fraction); UL, Unlabeled (intracellular fraction). #*P* < 0.05 vs. Control, *P < 0.05 vs. T1Dx. Statistical test: 1-way ANOVA, two-tailed *t*-test.

Once we established that insulin-deficient T1Dx animals were vulnerable to AF induction, which was rescued by insulin treatment, we then quantified cell surface and total protein expression of two major insulin-sensitive GLUT isoforms in the atria. Our results indicated a significant downregulation of total protein expression of both GLUT-4 and -8 in total lysate of atria of the T1Dx subjects compared to age-matched controls (by 24 and 38%, respectively, *P* = 0.011 and *P* = 0.01, respectively, Figures 3A,B), which was restored in the insulin-treated T1Dx group. In order to assess GLUT translocation to the atrial cell surface, the rate-limiting step in glucose uptake, we used the biotinylated photolabeling assay in the intact perfused heart to quantify active cell-surface GLUTs. We reported a significant downregulation of atrial cell surface GLUT-4 and -8 in the T1Dx animals compared to controls (by 67 and 80%, respectively, *P* < 0.001, Figures 3C,D) which was significantly rescued in the treated T1Dx subjects (*P* = 0.001 and *P* = 0.034, respectively, Figures 3C,D).

### Absence of Atrial Fibrosis and Glycogen Accumulation in the Atria of Untreated and Treated Diabetic Animals

**Figure 4:**
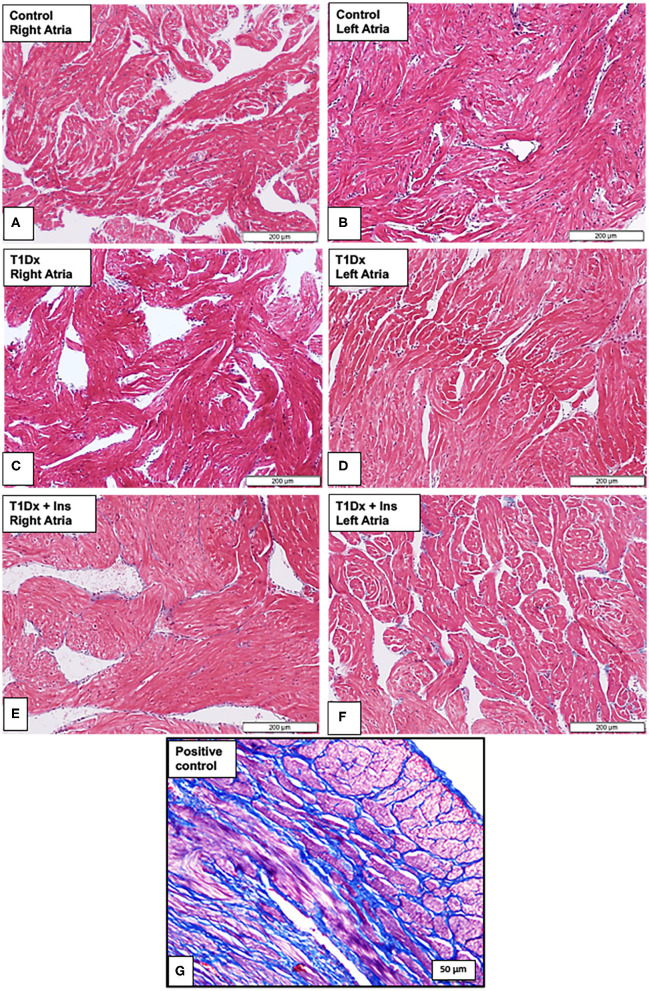
Absence of fibrosis in the atria of insulin-deficient type 1 diabetic (T1Dx) and insulin-treated diabetic (T1Dx+Ins) animals. Representative Masson's trichrome staining demonstrating the absence of fibrosis (blue staining fibrotic deposits) in **(A,B)** right and left atria of control animals; **(C,D)** right and left atria of T1Dx; **(E,F)** right and left atria of T1Dx+Insulin animals, scale bar: 200 μm. Control: *n* = 7, T1Dx: *n* = 7, and T1Dx+Ins: *n* = 3; **(G)** Positive control for Masson's trichrome staining (canine uterus, blue stain, scale bar: 50 μm).

**Figure 5:**
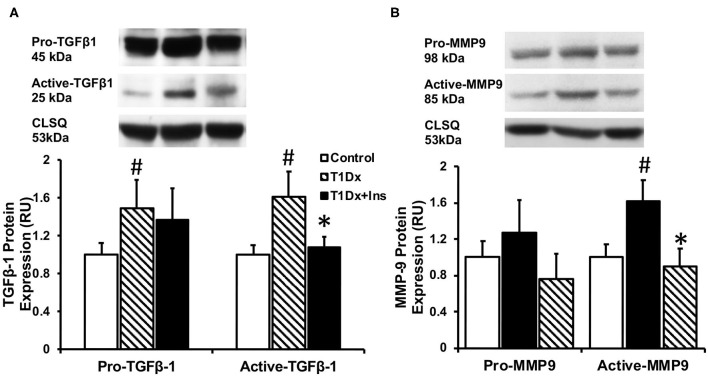
Increased protein expression of **(A)** active Transforming Growth Factor β-1 (TGFβ-1) and **(B)** active Matrix Metallo-Proteinase-9 (MMP-9) in the atria of type 1 diabetic (T1Dx) animals which was rescued in insulin-treated diabetic (T1Dx+Ins) animals. Top panel: representative Western blot from total lysate; loading control: calsequestrin (CLSQ). Bottom Panel: Mean ± SE of protein expression of pro- (latent) and active-TGFβ-1 and MMP9 (values normalized to respective controls); Control: *n* = 9, T1Dx: *n* = 9, and T1Dx+Ins: *n* = 4; #*P* < 0.05 vs. Control; *P < 0.05 vs. T1Dx; Methods: Western blotting. Statistical test: 1-way ANOVA.

Since atrial remodeling has been established as a precursor of AF (34–36), we determined whether atrial fibrosis could be involved in the pathogenesis of AF in our model. To this end, Masson's trichrome staining was used to assess the presence of fibrosis in the atria of untreated and insulin-treated diabetic animals, and age-matched control groups (15). Our results did not reveal any fibrosis in the atrial tissue sections of any groups (Figure 4). We then quantified the protein expression of the pro- (latent) and active- transforming growth factor −1 (TGFβ-1, a pro-fibrotic marker), and matrix metallo-proteinase-9 (MMP-9, an activator of TGFβ-1) in the total atrial tissue using Western blotting. A significant increase in the expression of pro and active TGFβ-1 was observed in the total lysate of atrial tissue of the T1Dx animals (by 49 and 61%, respectively, *P* = 0.026 and *P* = 0.025, respectively; Figure 5A) compared to controls. The upregulated expression of active TGFβ-1 was rescued in the atria of the T1Dx+Ins group (*P* = 0.043). Similarly, a significant up-regulation in the active form of MMP-9 was observed in T1Dx animals vs. age-matched controls (by 61%, *P* = 0.041), which was significantly rescued by *in vivo* insulin treatment (*P* = 0.044, Figure 5B). These findings indicate that while fibrosis is absent in the atria of the T1Dx animals, these insulin-deficient animals display a pro-fibrotic protein expression profile that was rescued by insulin treatment.

**Figure 6:**
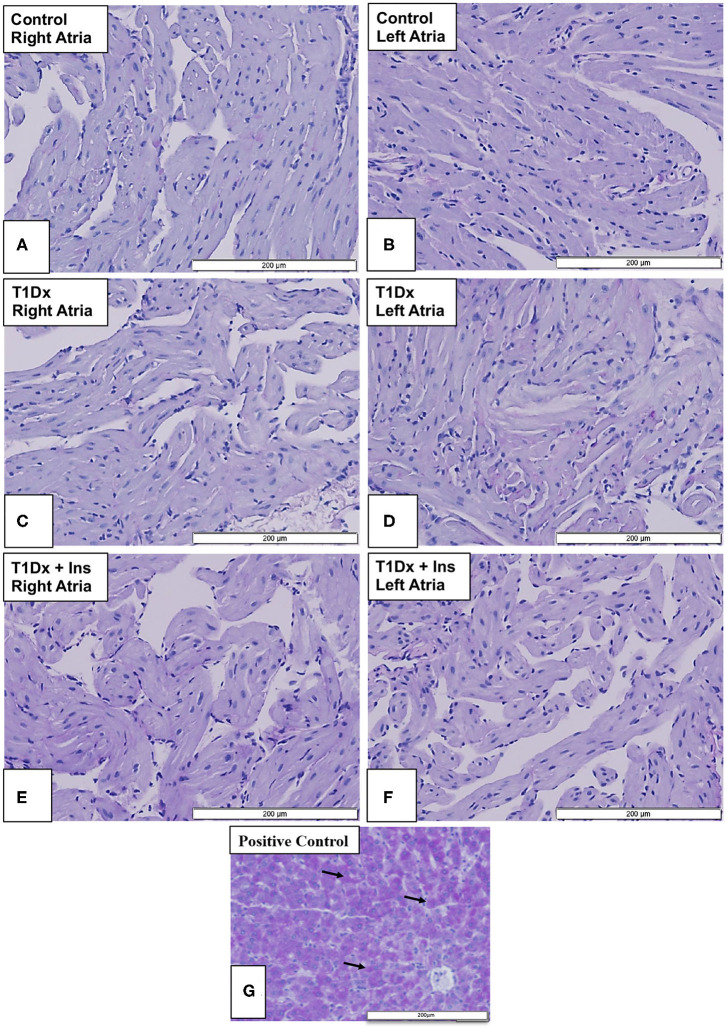
Absence of glycogen accumulation in the atria of insulin-deficient type 1 diabetic (T1Dx) and insulin-treated diabetic (T1Dx+Ins) animals. Periodic-acid Schiff (PAS) Stain (light purple indicating a negative result) in **(A,B)** right and left atria of control animals; **(C,D)** right and left atria of T1Dx; **(E,F)** right and left atria of T1Dx+Ins animals; **(G)** positive control for PAS staining (liver, dark purple, black arrows indicate positively-stained hepatocytes); scale bar: 200 μm; Control: *n* = 7, T1Dx: *n* = 7, and T1Dx+Ins: *n* = 3.

Impairment in glycolytic capacity may lead to abnormal glycogen accumulation in atrial tissue. However, we did not identify any glycogen deposits (using periodic acid-Schiff staining) in the atrial tissue in any of the groups (Figure 6).

## Discussion

The novel findings of this study included that: (1) insulin-deficient diabetic mice with mild hyperglycemia had an increased vulnerability to AF induction; (2) restoration of normo-glycemia in T1Dx animals by *in vivo* insulin treatment reduced AF vulnerability and propensity; (3) insulin-deficient diabetic mice displayed impairment in the expression and translocation of the major insulin-sensitive GLUT isoforms in the atria, which was rescued by *in vivo* insulin treatment.

Recently, we reported that insulin resistance during pre-diabetes and obesity significantly increased AF inducibility, including spontaneous AF (15). However, the pathogenic role of insulin deficiency during AF remains elusive and warrants further investigation. Therefore, in the present study, we used an insulin-deficient diabetic animal model, which also allows us to differentiate the complications resulting from the hyperglycemic state vs. obesity. Indeed, numerous studies have shown that obesity in itself (particularly obesity stemming from a high-fat diet) can cause several confounding variables, including inflammation, insulin resistance, hypertension, dyslipidemia, atherosclerosis, fatty liver disease, and kidney failure (37). Most importantly, obesity can often lead to an accumulation of epicardial and pericardial fat deposition, which has been reported to directly impact cardiac electrophysiology and contractility (38, 39), and lead to AF (40). Thus, it is pertinent to explore the potential causation of AF due to hyperglycemia alone, without the confounding complications of obesity. In the current study, we used an STZ model, which is one of the most common models to investigate complications associated with insulin deficient type 1 diabetes (29, 31). However, one of the limitations of this model is that it usually induces acute severe hyperglycemia without mimicking the autoimmune pathology of type 1 diabetic patients. Therefore, since there is a relationship between the STZ dose and the severity of diabetes, we previously established in our laboratory a protocol to induce a mild form of diabetes in order to mimic the early cardiac events that occur in diabetic subjects (31). Indeed, we have previously shown that there were no systolic and diastolic alterations in this diabetic model (31). However, echocardiographic evaluation of the atria in this model should be performed in future studies. In addition, in the present study, we investigated the effect of different glycemic level (i.e., normoglycemia, mild hyperglycemia, and restored euglycemia following long term *in vivo* insulin treatment) on the vulnerability to AF induction. However, future studies could be considered using glucose infusion or different levels of insulin dosing to correlate a wider range of glycemic levels with AF susceptibility. Finally, in order to control for any confounding variables associated with STZ administration itself, we used STZ-induced diabetic mice treated with insulin and demonstrated that all alterations were rescued in the insulin-treated mice, indicating that AF induction was not induced by potential STZ toxicity.

Although induction of AF in mice has been quite challenging due to the small size of the atria (limiting the formation of re-entry circuits, and thus fibrillatory activity) combined with the use of anesthesia (which may decrease AF propensity), we successfully induced AF in our mouse model using transesophageal atrial pacing in closed chest anesthetized control, untreated and treated diabetic animals, as previously described (33). While pacing of atria under anesthesia is considered a standard technique when examining propensity toward AF in both animal models and human patients (36, 41), a study in free-running mice fitted with telemetric monitoring would be an interesting future study. Although not all the animals were inducible, our results indicated that untreated T1Dx animals had overall significantly greater frequency and longer duration of atrial tachy-arrhythmias, including AF, atrial tachycardia and brady-tachy syndrome (which has been reported to precede AF) (42). Specifically, the frequency and duration of AF episodes were greater in T1Dx animals vs. controls. Few studies have investigated whether pharmacological interventions to correct hyperglycemia could reduce the AF burden. In the present study, insulin-treated diabetic mice remained protected against atrial tachy-arrhythmias, including AF. Therefore, our data demonstrates that insulin treatment partially protected insulin-deficient diabetic mice against AF susceptibility. In agreement with our findings, improvement in glycemic balance by insulin has been reported to decrease AF incidence during cardiac surgery (43). In addition, in a case report, treatment with rosiglitazone caused the regression of paroxysmal AF in diabetic patients (44). It has also been proposed that fluctuations in the glycemic state can initiate AF during diabetes rather than the prolonged hyperglycemic state by itself. Saito et al. demonstrated that glucose fluctuations increase the risk of AF in insulin-deficient diabetic rats (45). Therefore, we investigated the relationship between glycemic variability (i.e., hyperglycemia vs. normoglycemia) in type 1 diabetic mice and AF propensity. Restoration of euglycemia following insulin treatment was associated with reduced frequency of AF in diabetic mice. We further reported that AF vulnerability was significantly associated with blood glucose levels. In agreement with our findings, intra- and inter-atrial electromechanical delay has been positively correlated with fasting glucose levels (46). Although correlations between glucose levels and AF episodes are only associative in the present study, our findings suggest a novel role of insulin deficiency and impaired glucose metabolism in the pathogenesis of AF.

Recent studies have shown that progression of AF was associated with altered glucose transport and glycolytic inhibition (47). The heart expresses GLUT isoforms of class I, II, and III (e.g., GLUT-1, -3, -4, -8, -10, -11, and -12) (19, 30). Importantly, our laboratory reported that similar to the major cardiac isoform GLUT4, GLUT8 is an insulin-sensitive novel GLUT isoform in the healthy myocardium, including in the atria (19). We also reported that type 1 diabetes impairs the trafficking of both GLUT-4 and -8 to the atrial cell surface, which was correlated to blood glucose levels (19). We have likewise recently demonstrated that dysregulation of GLUT-4 and -8 trafficking is associated with AF propensity during insulin resistance and obesity (15). However, it remains debatable whether altered glucose metabolism plays a direct role in the pathogenesis of AF (48). Additionally, we recently demonstrated that *in vitro* stimulation of insulin restored GLUT trafficking in the atria of type 1 diabetic rodents (19). Therefore, we here capitalized on our recent findings to determine whether the restoration of atrial glucose transport following long-term *in vivo* insulin treatment will reduce the AF burden in insulin-deficient diabetic animals. Our results indicated a significant decrease in both total and active cell surface GLUT protein expression in the atria of T1Dx (vs. controls), which was rescued by long term *in vivo* insulin treatment. While pathophysiological mechanisms underlying AF remain to be investigated, one could speculate that restoration of GLUT protein expression and trafficking by *in vivo* insulin treatment restores glucose transport and reduces the glycolytic inhibition in atrial tissue, thereby providing a protective role against AF induction. Together, these findings suggest that insulin dysregulation and impaired glucose transport, secondary to altered GLUT trafficking, contribute to the overall pathogenesis of AF, although a causal link could not be provided in this study. Therefore, the pathophysiological mechanisms underlying AF during metabolic disease require further investigation, including the protein expression and function of ion channels in the atria. Indeed, previous studies indicated a variety of alterations of the generation and conduction of electrical signals in the hyperglycemic heart (49–51). Using similar diabetic animal models, we previously demonstrated that sustained outward K^+^ current and peak outward component of the inward rectifier were reduced in ventricular myocytes, while transient outward current was increased (49). In addition, Ca^2+^ transient amplitude was reduced and transient decay was prolonged in diabetic compared with control ventricular myocytes (50, 51). Finally, Howarth et al. found that the action potentials in the sinoatrial node of STZ-induced diabetic rats were significantly altered compared to controls, suggesting the potential pathogenic role of ion channel alterations during STZ-induced AF (52).

We further investigated potential additional metabolic mechanisms underlying increased arrhythmogenicity in our diabetic model. It has been suggested that glycogen accumulation in the diabetic atria impedes cell-to-cell conduction leading to enhanced atrial arrhythmogenicity, although results are controversial (23, 24, 53). For instance, we recently reported the absence of glycogen deposits in atrial tissue of insulin-resistant mice which had greater propensity for AF induction (15). In light of these recent findings, we investigated whether glycogen accumulation could occur in the atria of insulin-deficient type 1 diabetic animals. In contrast with previous studies that reported increased glycogen accumulation in the ventricle of diabetic patients and rodents (25–27), our results did not indicate any distinct location or distribution of glycogen in the atria of T1Dx animals compared to their respective controls. Similarly, in a diabetic rodent model (using high-fat diet with low dose STZ), it was reported that increasing STZ dose did not induce glycogen accumulation in the myocardium compared to controls (54). Therefore, findings from the present study suggest that in our mild diabetic animal model, glycogen deposition is not a contributing pathogenic factor during insulin deficiency-induced AF. Structural remodeling, including atrial fibrosis, has been identified as an important precursor of AF (23, 24, 53). Fibrotic deposits have been identified as potential anchoring points for reentry arrhythmias and attenuating forward wave propagation in the atrium and thereby facilitating the development of AF (53). In the present study, we did not observe any fibrotic deposits in our AF-inducible type 1 diabetic model, in agreement with previous findings in an insulin-resistant obese mice vulnerable to AF induction (15). Watanabe et al. reported that fibrotic deposits were observed in the atria of rats 16 weeks following STZ treatment (55). Therefore, our animal model used may not encapsulate all of the chronic alterations observed in clinical settings (56) and one could speculate that atrial fibrosis would have occurred if the duration of diabetes was prolonged or in a model with more severe hyperglycemia. Indeed, the overexpression of pro-fibrotic markers, such as TGFβ-1 and MMP-9, initiates atrial fibrosis and could contribute to atrial structural remodeling. Similarly, Nakano et al. reported significant increase in MMP-9 levels in atrial biopsies of human patients with both paroxysmal and persistent AF (57). In addition, increased activity of MMP-9 has been observed following rapid atrial pacing in both human and animal subjects (58, 59). Furthermore, it has been reported that MMP-9 can activate TGFβ-1 and facilitate atrial fibrosis (60). In addition, selective overexpression of TGFβ-1 in the atrium was sufficient to increase AF inducibility in both mice and transgenic goats (36, 61). Together, these results indicate a strong correlation between enhanced MMP-9 and TGFβ-1 expression and AF propensity. We have also previously reported increased expression of these pro-fibrotic markers during insulin resistance-induced AF (15). Similarly, our results demonstrate increased protein expression of active TGFβ-1 and MMP-9 in the atria of T1Dx animals, but not the insulin-treated diabetic animals, suggesting a potential link between the activation of these pro-fibrotic markers and the pathogenesis of AF.

In conclusion, using transesophageal atrial pacing, we demonstrated that insulin-deficient diabetic animals with mild hyperglycemia are at an increased risk of AF induction. In addition, AF burden of diabetic animals was reduced when both hyperglycemia and impaired atrial GLUT trafficking was rescued by *in vivo* insulin treatment. These findings suggest that alterations in glucose transport in the atria, due to the downregulation insulin-sensitive GLUT trafficking, is a pathophysiological factor of AF during type 1 diabetes. Because of the presence of the pacemaker in the atria, adequate glucose uptake, and utilization is critical for proper atrial function. It can be speculated that reduced cardiac glucose uptake due to the dysregulation of major insulin-sensitive GLUT isoforms (e.g., GLUT-4 and -8) would lead to an overall reduction in ATP production which could potentially cause abnormal function of major cardiac ion pumps (e.g., SERCA pump and the membrane bound Ca^2+^ ATPase pump) (62). This is germane to the fact that alteration of inward calcium current and subsequent shortening of the action potential duration of atrial myocytes are pathogenic factors of AF (49, 63). However, additional studies measuring glucose oxidation and ATP production in diabetic hearts will be required to test this hypothetical mechanism. Overall, the results obtained from this study suggested that altered glucose transport in the atria, due to insulin deficiency and subsequent mild hyperglycemia, could be an early metabolic pathogenic factor of AF during diabetes. However, additional studies are required to study the role of altered glucose homeostasis leading to electrophysiological disturbances and fibrillation in the atria of diabetic subjects.

## Data Availability Statement

All data generated for this study are included in the article.

## Ethics Statement

The animal study was reviewed and approved by the Oklahoma State University Institutional Animal Care and Use Committee (animal protocol # VM-12-3).

## Author Contributions

VL conceived and designed the experiments. ZM, AC, and BS performed the experiments. ZM, AC, BS, JR, and VL analyzed and interpreted the data. VL, ZM, AC, BS, and JR wrote and/or edited the manuscript. All authors contributed to the article and approved the submitted version.

## Conflict of Interest

The authors declare that the research was conducted in the absence of any commercial or financial relationships that could be construed as a potential conflict of interest.
